# Endotrophin is a risk marker of complications in CANagliflozin cardioVascular Assessment Study (CANVAS): a randomized controlled trial

**DOI:** 10.1186/s12933-022-01666-7

**Published:** 2022-11-28

**Authors:** Daniel Guldager Kring Rasmussen, Michael K. Hansen, Joseph Blair, Timothy A. Jatkoe, Bruce Neal, Morten A. Karsdal, Federica Genovese

**Affiliations:** 1 Nordic Bioscience A/S, Herlev, Denmark; 2 Janssen Research & Development, LLC, Spring House, Raritan, PA USA; 3 Janssen Research & Development, LLC, Raritan, NJ USA; 4 The George Institute for Global Health, UNSW Sydney, Sydney, Australia; 5 The Charles Perkins Centre, University of Sydney, Sydney, NSW Australia; 6 Imperial College London, London, UK

**Keywords:** Biomarker, Collagen, Diabetes, Endotrophin, Extracellular matrix, Fibrosis, Heart failure, Mortality, Prognostic

## Abstract

**Background:**

Enhanced de-novo collagen type VI (COL VI) formation has been associated with kidney and cardiovascular fibrosis. We hypothesized that endotrophin (ETP), a product specifically generated during collagen type VI formation, may be prognostic for heart failure (HF), cardiovascular death (CVD), kidney endpoints, and all-cause mortality in patients with type 2 diabetes.

**Methods:**

We measured ETP in plasma (P-ETP) and urine (U-ETP) samples collected at baseline and follow-up (year 3) from the randomized controlled trial, CANagliflozin cardioVascular Assessment Study (CANVAS), by use of the PRO-C6 ELISA measuring COL VI formation and ETP. At baseline, plasma and urine samples were available for 3531 and 3423 patients, respectively. At year 3, plasma and urine samples were available for 2178 (61.7%) and 2070 (60.5%) patients, respectively Patients were followed for a median of 6.1 years, and endpoints included: incident HF, CVD, three kidney composite endpoints, and all-cause mortality. Backward selection was used to identify variables to be included in the analyses. Robustness of the association with outcome was assessed by bootstrap analyses.

**Results:**

In univariable analysis, P-ETP predicted all investigated outcomes (all p < 0.0001), remained independently associated with all outcomes after adjustment for conventional risk factors (all p < 0.004), and increased C-statistics of the models for the outcomes HF, CVD, HFCVD, all-cause mortality, and kidney composite 2 (ΔC ≥ 0.002). In bootstrap analysis, P-ETP was retained with a frequency ranging from 41.0 to 98.4% for all outcomes. Levels of U-ETP were associated with outcomes in univariable analysis, but associations with most outcomes were lost after adjustment for conventional risk factors. The increase in P-ETP over time was greater with increasing albuminuria stage (p < 0.0001) and was independently associated with the kidney endpoints (p < 0.03). In the placebo arm, the increase in P-ETP was prognostic for all-cause mortality (HR [95% CI]; 1.14 [1.05–1.23], p = 0.003). Whereas levels of P-ETP were not impacted by treatment, levels of U-ETP significantly increased with canagliflozin treatment.

**Conclusions:**

P-ETP generated during COL VI formation predicts cardiovascular, kidney and mortality outcomes in patients with type 2 diabetes. As ETP identifies patients at increased risk of experiencing relevant outcomes, it may be used for patient enrichment in future clinical trials.

*Trial Registry Number* (ClinicalTrials.gov Identifier): NCT01032629

**Supplementary Information:**

The online version contains supplementary material available at 10.1186/s12933-022-01666-7.

## Research in context

- **What is already known about this subject? (maximum of 3 bullet points)**
  - Patients with type 2 diabetes are at increased risk of experiencing cardiovascular and kidney outcomes, and mortality
  - Canagliflozin has been suggested to have anti-inflammatory and anti-fibrotic effects
  - Endotrophin (ETP) has been shown to be prognostic for outcomes in a small observational study of patients with type 2 diabetes
- **What is the key question? (one bullet point only; formatted as a question)**
  - Is ETP associated with increased risk of experiencing clinically relevant outcomes in a clinical trial population?
- **What are the new findings? (maximum of 3 bullet points)**
  - ETP is a robust, independent prognostic marker for predicting outcome in a clinical trial population of patients with type 2 diabetes
  - ETP is not affected by canagliflozin treatment in patients with type 2 diabetes
  - An increase in ETP is associated with increased risk of all-cause mortality
- **How might this impact on clinical practice in the foreseeable future? (one bullet point only)**
  - ETP levels may be used to enrich clinical trials for patients at risk of outcome. In line with this, the FDA has issued a Letter of Support for the prognostic marker PRO-C6 (ETP) in the setting of heart failure.

## Introduction

Type 2 diabetes is the most common etiology in patients with chronic kidney disease (CKD). Diabetic patients are prone to develop moderately increased albuminuria which is associated with an accelerated deterioration of kidney function [1]. Sodium glucose co-transporter 2 (SGLT2) inhibitors lower blood glucose in patients with type 2 diabetes by increasing urinary glucose excretion, which also results in a mild osmotic diuresis and a net caloric loss [2]. In the CANagliflozin cardioVascular Assessment Study (CANVAS) Program, among patients with type 2 diabetes who had an increased risk of cardiovascular (CV) disease, patients treated with canagliflozin (CANA) had a lower risk of cardiovascular and renal outcomes than those who received placebo (PBO) [3]. The mechanism of renal and CV benefit associated with SGLT2 inhibition is unclear and likely has substantive effects beyond glucose lowering [4–9]. It has been proposed that SGLT2 inhibition reduces inflammation in the tissues, and that it may therefore impact the turnover of the extracellular matrix (ECM). Regardless of the underlying etiology, the progression of kidney disease is assumed to be driven by kidney fibrosis caused by an abnormal shift in the turnover of components of the kidney ECM [10, 11]. Collagen is an essential part of the fibrotic structure acting as a scaffold for a range of interaction partners and cell-adhesion [12–14]. Patients with active kidney fibrosis are at higher risk for cardiovascular outcomes, mortality, and rapid deterioration of kidney function [15]. During fibrosis development, collagen molecules are produced in excess. It is becoming increasingly clear that the well-organized architecture of the ECM not only provides a structural scaffold, but that it also actively engages in signaling by releasing fragments with signaling properties. One fragment that has received increasing attention is endotrophin, a fragment released from the alpha-3 chain of COL VI. Endotrophin (ETP) has been shown to possess both pro-inflammatory and pro-fibrotic properties, enhancing TGF-β signaling, attracting macrophages, and promoting epithelial-to-mesenchymal transition [16, 17]. The PRO-C6 assay detects the propeptide of COL VI released during COL VI formation [18, 19], which contains the signaling fragment ETP [16, 17]. Consequently, PRO-C6 reflects both COL VI formation and the amount of released ETP.

There are limited data on the effects of SGLT2 inhibitors on the ECM. Previous studies in patients with both type 1 and 2 diabetes have revealed that ETP is able to predict kidney and cardiovascular outcome [20, 21]. In our study we tested whether ETP predicts CV and kidney endpoints and all-cause mortality in CANVAS. We also assessed longitudinal changes in the concentrations of ETP in patients with type 2 diabetes receiving CANA or PBO. We assessed levels of ETP in both urine and plasma, as urinary levels are more likely to reflect alterations to the kidney, whereas levels in plasma are more likely to reflect systemic changes, such as alterations to the cardiovascular system.

## Research design and methods

### Study population

**Fig. 1:**
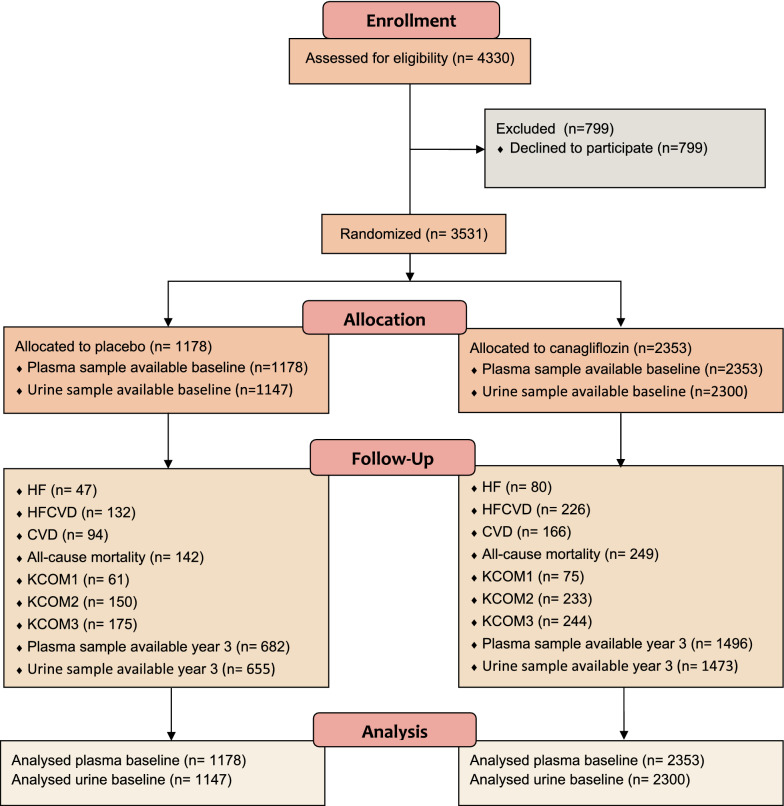
Flow diagram of CANVAS subset used for the current analysis. The subset of CANVAS used in the current analysis consisted of patients that agreed to samples being used for research (n = 3531). Of the 3531 patients who gave permission, baseline plasma samples were available for 3531 patients (1178 placebo/2353 CANA) and baseline urine samples were available for 3447 patients (1147 placebo/2300 CANA). At year 3, plasma samples were available for 2178 patients (682 placebo/1496 CANA), and urine samples were available for 2128 (655 placebo/1473 CANA). The discrepancy in number between plasma and urine samples was due to missing samples

This post hoc, exploratory analysis was conducted using a subset of stored plasma samples from the CANVAS trial (ClinicalTrials.gov identifier: NCT01032629), which was a randomized, multicenter, double-blind, parallel, PBO-controlled study that evaluated the efficacy and safety of CANA 100 and 300 mg (1:1:1 allocation). The inclusion and exclusion criteria have been described in previous publications [22]. In short, inclusion criteria were diagnosis of type 2 diabetes and age ≥ 30 years with a history of a CV event or age ≥ 50 years with a high risk of CV events, inadequate diabetes control (as defined by glycated hemoglobin [HbA1c] ≥ 7.0% to ≤ 10.5% at screening) and either (1) not currently on diabetes drug therapy or (2) on therapy with any approved class of diabetes drugs. Exclusion criteria were history of diabetic ketoacidosis, type 1 diabetes, pancreas or beta-cell transplantation, diabetes secondary to pancreatitis or pancreatectomy, history of ≥ 1 severe hypoglycemic episode within 6 months before screening. Median (range) follow-up time was 6.1 (6.1–7.1) years. A flow diagram for the used patient population is shown in Fig. 1.

### Measurement of ETP by enzyme-linked immunosorbent assay (ELISA)

Baseline plasma samples from 3531 patients and urine from 3423 patients were available for this study. At year 3, plasma and urine samples were available for 2178 (61.7%) and 2070 (60.5%), respectively. ETP was measured in plasma (P-ETP) and urine (U-ETP) using a previously described competitive enzyme-linked immunosorbent assay, namely PRO-C6, developed by Nordic Bioscience, Denmark [21]. The unit for P-ETP is ng/mL, whereas the unit for U-ETP is ng/mmol creatinine.

### Outcomes

The investigated outcomes have been described previously [22]. The outcomes investigated included hospitalization due to heart failure (HF), cardiovascular death (CVD), a composite of HF and CVD (HFCVD), and all-cause mortality. The first composite kidney endpoint (KCOM1) was defined as a sustained 40% decline of eGFR, end-stage kidney disease defined as an eGFR < 15 mL/min/1.73 m^2^, need for dialysis or kidney transplantation, or kidney death. The second kidney composite endpoint, KCOM2, included KCOM1 and CVD, and the third kidney composite endpoint, KCOM3, included KCOM1 and conversion to severely increased albuminuria.

### Statistical analyses

Given the similar treatment effect from both canagliflozin treatment regimens (100 and 300 mg daily), data from patients receiving either of the 2 doses were combined in this analysis.

Associations between ETP and relevant variables were investigated using non-parametric Spearman-rank correlations.

To investigate which variables were independently associated with ETP, we used multivariable regression analysis with P-ETP as the dependent variable and age, sex, body mass index (BMI), diabetes duration, HbA1c, low density lipoprotein (LDL), high density lipoprotein (HDL), diastolic and systolic blood pressure (BP), eGFR, albumin:creatinine ratio (ACR), AST, ALT, and NTproBNP as the independent variables. The association of these variables with P-ETP were reported as r_partial_.

A Mann–Whitney test was used to assess whether levels of P-ETP were elevated in patients with prior history of HF and prior history of CV disease. To assess whether P-ETP was higher in these patients independent of clinical covariates, a multivariate logistic regression analysis was performed with P-ETP, and the clinical covariates age, sex, BMI, diabetes duration, HbA1c, LDL, HDL, diastolic and systolic BP, eGFR, and ACR, AST, ALT, and NTproBNP.

Unadjusted Kaplan–Meier curves were used to visualize the association of tertiles of P-ETP and U-ETP with the investigated end-points.

Absolute change from baseline in plasma and urine levels of ETP (Nordic Bioscience, Herlev, Denmark) were analyzed in patients with data available at both baseline and Week 156 (n = 2178 and n = 2070, respectively). The absolute change for P-ETP (ΔP-ETP) and U-ETP (ΔU-ETP) was calculated as year 3 minus baseline ETP in plasma and urine, respectively. The unit for ΔP-ETP was ng/mL, whereas the unit for ΔU-ETP was ng/mmol.

We performed both univariable and multivariable C_ox_ proportional hazard regression analysis. Treatment was included as an interaction term in all outcome analysis to assess whether there were differences in biomarker performance between placebo and canagliflozin treated patients. If no differences were observed, results from the combined treated arms were shown.

Backward selection using the Akaike information criterion (AIC) was employed to identify variables retained in a Cox proportional hazards model for each outcome. The variable input in the model build were age, sex, BMI, systolic BP, diastolic BP, HbA1c, diabetes duration, LDL, ACR, eGFR, AST, ALT, NTproBNP, prior history of HF, prior history of CV disease, treatment, smoking, and either P-ETP or U-ETP. To assess the robustness of the selected model, backwards selection was performed in 500 bootstrap iterations with random resampling. Hazard ratios (HRs) for ETP were presented adjusted for the clinical parameters included in the final model. The HRs for ETP at baseline were reported per doubling in biomarker levels (i.e., log2–transformation). As negative values were seen for the ΔP-ETP and ΔU-ETP, HRs for these analyses are presented per increments of 1 ng/mL and ng/mmol, respectively.

An ANOVA model with Tukey’s multiple comparisons test was used to determine overall differences in ΔP-ETP and ΔU-ETP levels between albuminuria stages.

Parametric analysis was performed for data having a Gaussian distribution (normally distributed) and non-parametric analysis was performed for data having a non-Gaussian distribution (non-normally distributed). Distribution of the data was assessed by the D’Agostino and Pearson normality test.

All 2-tailed p values of < 0.05 were considered significant. Statistical analyses were made by the MedCalc statistical software (MedCalc, Belgium), SAS software (version 9.4, SAS Institute, Cary, NC, USA) or R studio (Version 1.4.1106), and visualized using GraphPad Prism version 9 (GraphPad Software, San Diego, CA, USA).

## Results

The demographics, laboratory results, and clinical history are presented for all patients with plasma samples available at baseline in patients of the CANVAS biomarker sub-study (Table 1). The total population had an average (± SD) age of 62.8 (± 7.9) and 33.2% were female. With an average BMI of 32.7 (± 6.1) patients were generally classified as obese. Patients had an average eGFR of 77.0 (± 18.8) ml/min/1.73 m^2^, and an ACR of 11.5 (6.4–34.7) mg/g. At baseline, patients were predominantly classified as having normal to mildly increased ACR (72.5%) with 21.9% having moderately increased, and 5.5% severely increased, ACR.

**Table 1:** Baseline demographic and disease characteristics among patients with samples available at baseline

Characteristic	CANA (n = 2353)	PBO (n = 1178)	Total (N = 3531)
Age, year	62.9 ± 7.9	62.6 ± 7.8	62.8 ± 7.9
Female sex, n (%)	779 (33.1)	392 (33.3)	1171 (33.2)
Current smoker, n (%)	410 (17.4)	242 (20.5)	652 (18.5)
History of hypertension, n (%)	2067 (87.8)	1041 (88.4)	3108 (88.0)
History of HF, n (%)	296 (12.6)	171 (14.5)	467 (13.2)
Duration of diabetes, year	13.6 ± 7.5	13.3 ± 7.6	13.5 ± 7.5
Prior history of CV disease, n (%)	1410 (59.9)	695 (59.0)	2105 (59.6)
Prior history of HF, n (%)	171 (14.5)	296 (12.6)	467 (13.2)
BMI, kg/m^2^	32.7 ± 6.1	32.6 ± 6.2	32.7 ± 6.1
Systolic BP, mmHg	136.3 ± 15.9	137.1 ± 15.7	136.6 ± 15.8
Diastolic BP, mmHg	77.4 ± 9.8	78.1 ± 9.8	77.6 ± 9.8
HbA1c, %	8.2 ± 0.9	8.1 ± 0.9	8.2 ± 0.9
HDL, mg/dL (Median [IQR])	44 [38–52]	44 [38–53]	44 [38–52]
LDL, mg/dL (Median [IQR])	88 [63–108]	82 [63–107]	82 [63–108]
AST, U/L	23.4 (9.8)	23.7 (12.2)	23.5 (10.6)
ALT, U/L	27.0 (14.8)	27.3 (13.9)	27.1 (14.5)
NTproBNP, ng/mL (Median [IQR])	90 [42–205]	96 [42–196]	92 [42–204]
eGFR, mL/min/1.73 m^2^	77.1 ± 18.8	76.7 ± 19.0	77.0 ± 18.8
ACR, mg/g (Median [IQR])	11.6 (6.5–34.4)	11.5 (6.2–36.0)	11.5 (6.4–34.7)
Normal/mildly increased, n/total (%)	1704/2341 (72.8)	844/1173 (72.0)	2548/3514 (72.5)
Moderately increased, n/total (%)	519/2341 (22.2)	252/1173 (21.5)	771/3514 (21.9)
Severely increased, n/total (%)	118/2341 (5.0)	77/1173 (6.6)	195/3514 (5.5)

Data are mean ± SD unless otherwise indicated

*SD* standard deviation, *HF* heart failure, *CV* cardiovascular, *BMI* body mass index, *BP* blood pressure, *HDL* high density lipoprotein, *LDL* low density lipoprotein, *AST* aspartate aminotransferase, *ALT* alanine aminotransferase, *NTproBNP* N-terminal pro-brain natriuretic peptide, *eGFR* estimated glomerular filtration rate, *ACR* albumin:creatinine ratio *IQR* interquartile range

### Association of ETP with variables in cross-sectional analysis

First, we investigated whether levels of ETP in plasma (P-ETP) or urine (U-ETP) correlated with demographic and clinical variables. We found that P-ETP had a very weak positive association with age (rho = 0.12, p < 0.0001), diabetes duration (rho = 0.15, p < 0.0001), BMI (rho = 0.17, p < 0.0001), HbA1c (rho = 0.06, p = 0.0005), NTproBNP (rho = 0.22, p < 0.0001), and ACR (rho = 0.13, p < 0.0001). P-ETP had a very weak negative association with diastolic blood pressure (rho = − 0.07, p = 0.0001), HDL (rho = − 0.05, p = 0.001), ALT (rho = − 0.08, p < 0.0001), and a weak to moderate negative association to eGFR (rho = − 0.39, p < 0.0001). There was no association between P-ETP and LDL or AST. Levels of P-ETP were higher in females (median [IQR], 9.5 [7.4–12.2] ng/mL) than in males (median [IQR], 8.9 [7.1–11.5], p < 0.0001). P-ETP was not different between ethnic groups. In a multivariable regression analysis with P-ETP as the dependent variable and the above-mentioned variables as independent variables, we found that sex (r_partial_ = − 0.05, p = 0.004), BMI (r_partial_ = 0.13, p < 0.0001), diabetes duration (r_partial_ = 0.07, p < 0.0001), HDL (r_partial_ = − 0.04, p = 0.01), HbA1c (r_partial_ = 0.05, p = 0.003), eGFR (r_partial_ = − 0.26, p < 0.0001), ACR (r_partial_ = 0.11, p < 0.0001), AST (r_partial_ = 0.07, p = 0.0001), ALT (r_partial_ = − 0.06, p = 0.0008), and NTproBNP (r_partial_ = 0.10, p < 0.0001) were independently associated with P-ETP.

We also explored levels of P-ETP in patients with prior history of HF and CV disease. P-ETP levels were significantly higher in patients with prior history of HF (median [IQR], 9.8 [7.4–12.3] vs 9.0 [8.9–9.2], p = 0.004) and prior history of CV disease (median [IQR], 9.3 [7.3–12.1] vs 8.9 [7.0–11.3], p = 0.0002). In multivariate logistic regression analysis including all investigated clinical covariates, P-ETP was no longer associated with either prior history of HF and prior history of CV disease (both p < 0.26).

U-ETP levels had a weak positive association with age (rho = 0.05, p = 0.003), diabetes duration (rho = 0.06, p = 0.0004), diastolic (rho = 0.05, p = 0.004) and systolic blood pressure (rho = 0.1, p < 0.0001), HbA1c (rho = 0.04, p = 0.01), LDL (rho = 0.07, p < 0.0001), HDL (rho = 0.13, p < 0.0001), eGFR (rho = 0.05, p = 0.006), ACR (rho = 0.11, p < 0.0001) and NTproBNP (rho = 0.10, p < 0.0001), and a weak negative association with AST (rho = − 0.07, p = 0.0001) and ALT (rho = − 0.09, p = 0.0001). U-ETP was significantly higher in hispanics or latino (p = 0.0009). U-ETP levels were not able to discriminate patients with prior history of HF (p = 0.25) but were significantly lower in patients with prior history of CV disease (median [IQR], 239.5 [185.0–324.0] vs 258.0 [194.0–347.3], p = 0.0001). In multivariate logistic regression analysis including all investigated clinical covariates, U-ETP was no longer associated with prior history of CV disease (p = 0.16).

### Association of ETP with outcomes

**Fig. 2:**
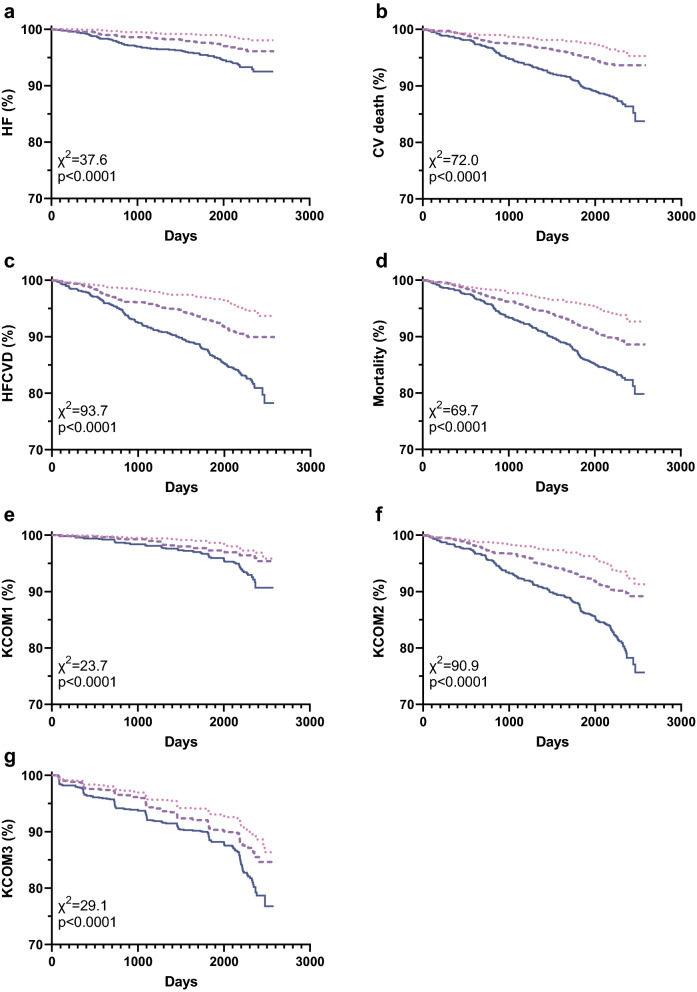
Association of P-ETP with outcome. Patients were stratified based on baseline P-ETP into tertiles and Kaplan–Meier curves were plotted for each tertile. Risk was assessed with reference to tertile 1 for (**a**) heart failure (HF), (**b**) CV death, (**c**) the composite of HF and CV death, (**d**) all-cause mortality, (**e**) the kidney composite endpoint 1 (KCOM1; 40% decrease in eGFR, kidney death, or ESKD), (**f**) -2 (KCOM2; KCOM1 and CVD), and (**g**) -3 (KCOM3; KCOM1 and conversion to severely increased albuminuria). Tertile 1, ; Tertile 2, ; and Tertile 3,

In univariable analysis for all investigated outcomes, patients with increasing levels of P-ETP had increased risk of experiencing an outcome (all p < 0.0001, Fig. 2 and Additional file 1: Fig. S1). In univariable analysis, patients with the highest levels of U-ETP in the urine were at increased risk of experiencing all of the investigated outcomes (all p < 0.01), except HF (Additional file 1: Fig. S2).

There was no interaction between P-ETP and treatment arms for any of the investigated outcomes (all p > 0.23). We therefore looked at all patients in a pooled analysis. We used Cox proportional hazard regression analysis to investigate the prognostic ability of P-ETP and U-ETP to predict the investigated outcomes in all patients. In univariable analysis, P-ETP predicted all outcomes (all p < 0.0001). When adjusted for conventional risk factors, P-ETP was independently associated with all of the investigated outcomes (all p < 0.008; Table 2). Addition of P-ETP to the model of conventional risk factors improved the C-index for all endpoints except the kidney composite endpoints KCOM1 and KCOM3 (Table 2).

**Table 2:** Association of P-ETP with Outcomes in the CANVAS Study

	Variables retained	HR_adj_ (95% CI) for P-ETP^a^	P	C index (clinical covariates)	C index (P-ETP)	C index combined (∆C)
HF	P-ETP, age, sex, BMI, HbA1c, dBP, sBP, ACR, NTproBNP, prior history of HF, prior history of CV disease	1.57 [1.15–2.14]	0.005	0.823	0.679	0.828 (0.005)
CVD	P-ETP, age, LDL, ACR, NTproBNP, prior history of HF, prior history of CV disease	1.52 [1.23–1.88]	0.0001	0.755	0.646	0.757 (0.002)
HFCVD	P-ETP, age, BMI, HbA1c, ACR, treatment, NTproBNP, smoking, prior history of HF, prior history of CV disease	1.49 [1.23–1.79]	< 0.0001	0.771	0.651	0.776 (0.005)
Mortality	P-ETP, age, BMI, dBP, LDL, ACR, NTproBNP, smoking, prior history of CV disease	1.42 [1.19–1.70]	0.0001	0.720	0.624	0.724 (0.004)
KCOM1	P-ETP, ACR, treatment, NTproBNP	1.54 [1.15–2.06]	0.004	0.774	0.631	0.773 (− 0.001)
KCOM2	P-ETP, age, BMI, LDL, ACR, treatment, NTproBNP, prior history of CV disease	1.45 [1.22–1.74]	< 0.0001	0.757	0.639	0.760 (0.003)
KCOM3	ACR, treatment	1.26 [1.06–1.48]^a^	0.008	0.778	0.583	0.777 (− 0.001)

HR_adj_, adjusted HR per doubling (log2) of P-ETP. Covariates in the initial model included age, sex, body mass index (BMI), diabetes duration, HbA1c, LDL, diastolic and systolic blood pressure (BP), eGFR, albumin:creatinine ratio (ACR), treatment, NTproBNP, smoking, prior history of HF, and prior history of CV disease

*KCOM1* comprised a 40% decrease in eGFR (sustained for two or more consecutive measures), the need for RRT (dialysis or transplantation), or renal death, *KCOM2* comprised KCOM1 and CVD, *KCOM3* comprised KCOM1 and progression to macroalbuminuria, *HF* heart failure, *BMI* body mass index, *dBP* diastolic blood pressure, *sBP* systolic blood pressure, *ACR* albumin:creatinine ratio, *CVD* cardiovascular death, *HFCVD* composite of HF and CVD, *KCOM* kidney composite endpoint

^a^Using the Akaike information criterion in backwards selection, covariates for adjustment of P-ETP were selected. The variables retained in the final model were listed in the column “Variables retained”. C index values are given for clinical covariates only (“C index (clinical covariates only)”, only P-ETP (“C index (P-ETP)”), combined (“C index combined”), and the improvement in the C index when adding P-ETP to the base model only including clinical covariates (“∆C”). ^a^Despite the significance, P-ETP was not retained in the final model after the bootstrap analyses

It has previously been shown that there was a significant association between P-ETP and liver disease severity. We therefore investigated whether further adjustment for liver injury markers (AST and ALT) impacted the association. Adjustment for these parameters did not alter the association of P-ETP with any of the outcomes (data not shown).

**Fig. 3:**
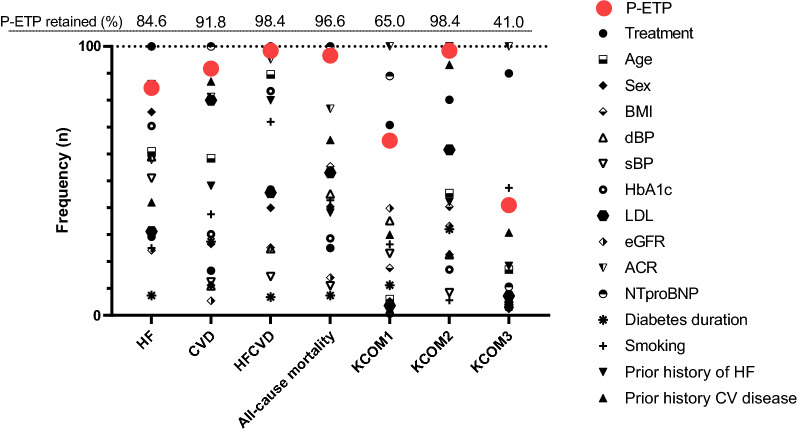
Frequency of biomarker retention in the final prognostic model. To test the robustness of the association with outcome, we performed 500 bootstraps with random resampling. For each of the iterations, backward selection using the Akaike information criterion (AIC) was used to identify the variables retained in the final model. Variables included in the base model before backward selection of each iteration were age, sex, BMI, systolic BP, diastolic BP, HbA1c, diabetes duration, low-density lipoprotein cholesterol (LDL), albumin: creatinine ratio (ACR), estimated glomerular filtration rate (eGFR), treatment, prior history of HF, prior history of CV disease, NTproBNP, smoking, and P-ETP. The proportion (%) of times that P-ETP was retained in the final model is listed at the top of the figure

To test the robustness of the association with outcome, we performed 500 bootstraps with random resampling of patients from CANVAS. For each of the iterations, backward selection using the Akaike information criterion (AIC) was used to identify the variables retained in the final model. Out of the 500 iterations, P-ETP was retained 423 times for HF (84.6%), 459 times for CVD (91.8%), 492 times for HFCVD (98.4%), 483 times for all-cause mortality (96.6%), 325 times for KCOM1 (65.0%), 492 times for KCOM2 (98.4%), and 205 times for KCOM3 (41.0%) (Fig. 3).

U-ETP was prognostic for the outcomes of HF, the composite HFCVD, KCOM1 and KCOM2 (all p < 0.05). In multivariable Cox proportional hazard regression analysis with backward selection, U-ETP was not retained in any of the final models for all outcomes (data not shown).

## Association of changes in ETP levels with treatment and outcome

**Fig. 4:**
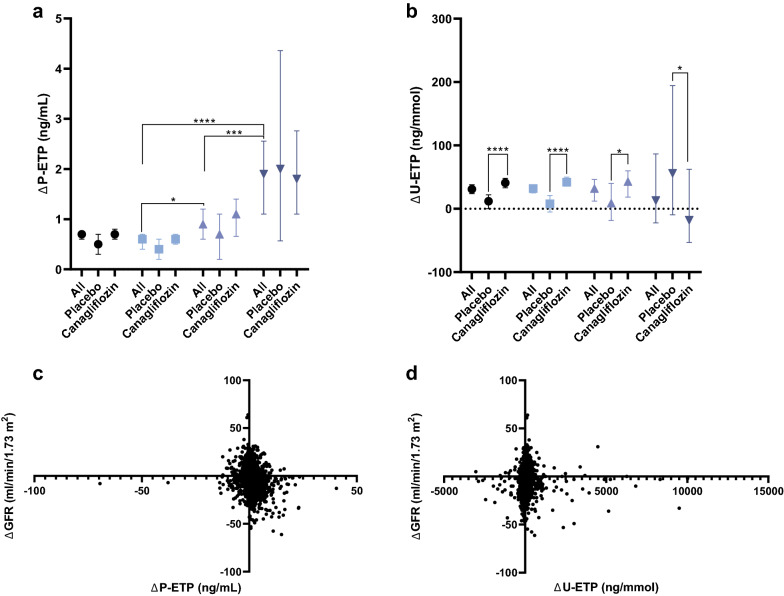
Association of ETP changes with albuminuria stages and changes in eGFR. **a** Compared to patients with normal to mildly elevated albuminuria, an increase in P-ETP (ΔP-ETP) was observed in patients with moderately elevated, which further increased in patients with severely elevated albuminuria. **b** No change in U-ETP (ΔU-ETP) levels were observed between patients stratified by albuminuria status, but levels were significantly higher in patients treated with canagliflozin. **c** Whereas there was a significant weak negative correlation between the change in P-ETP and eGFR over the study (rho = − 0.24, p < 0.0001), a significant very weak positive correlation was observed for (**d**) U-ETP (rho = 0.11, p < 0.0001). For graph (**a**) and (**b**), median and [95% CI] is plotted for all patients combined (), and stratified into normal to mildly (), moderately (), and severely elevated albuminuria ()

At year 3, plasma and urine samples were available for 2178 (61.7%) and 2070 (60.5%), respectively. Whereas treatment did not impact levels of P-ETP (Fig. 4a) there was a significant increase in U-ETP in the CANA treated group (p < 0.0001, Fig. 4b).

For each of the albuminuria stages, the increase in P-ETP (ΔP-ETP) was similar between the CANA and PBO arms (Fig. 4a). Despite levels of ΔP-ETP being similar between treatment arms, analysis in the overall cohort revealed that ΔP-ETP increased in patients with moderately elevated albuminuria compared to normal to mildly elevated albuminuria (p = 0.03), and further increased in patients with severely elevated albuminuria compared to patients with moderately elevated albuminuria (p < 0.001, Fig. 4a). There was no difference in the change of U-ETP (ΔU-ETP) between albuminuria groups (Fig. 4b).

A weak negative association was observed between ΔETP and ΔeGFR (rho = − 0.24, p < 0.0001; Fig. 4c), and a very weak positive association between ΔU-ETP and ΔeGFR (rho = 0.11, p < 0.0001; Fig. 4d). Despite the opposite trend between ΔP-ETP and ΔU-ETP with ΔeGFR, there was no correlation between ΔP-ETP and ΔU-ETP (rho = 0.03, p = 0.17).

Whereas no association was seen between the change in albuminuria and ΔP-ETP, a low association was seen between the change in albuminuria and change in U-ETP (rho = 0.13, p < 0.0001).

We found that ΔP-ETP was independently associated with the kidney composite endpoints (KCOM1 (HR [95% CI]; 1.08 [1.03–1.12], p = 0.0005), KCOM2 (HR [95% CI] 1.05 [1.02–1.09], p = 0.004), and KCOM3 (HR [95% CI] 1.03 [1.00–1.06], p = 0.03)). Interestingly, whereas there were no interactions between ΔP-ETP and treatment investigating the other outcomes, we found that there was an interaction between ΔP-ETP and treatment when looking at all-cause mortality (p = 0.008). Looking into this, we found that ΔP-ETP was associated with all-cause mortality in the PBO arm both unadjusted (HR [95% CI] 1.11 [1.05–1.18], p = 0.0004) and adjusted for variables associated with mortality (HR [95% CI]; 1.14 [1.05–1.23], p = 0.003).

## Discussion

In this subset of patients from the CANVAS trial, the COL VI marker ETP, which has shown prognostic value as a biomarker of CV risk in type 1 diabetes [20] and type 2 diabetes [21] was prognostic for cardiovascular outcomes, kidney outcomes and all-cause mortality independent of conventional risk factors. The robust association of P-ETP with the investigated endpoints was seen from the bootstrap analysis, where P-ETP was consistently retained in the final model even in the presence of other risk markers such as NTproBNP. Levels of P-ETP increased over a 3 year period in patients allocated to both PBO or CANA treatment. The increase in P-ETP levels over time was greater in patients with higher baseline albuminuria stage. U-ETP increased with CANA treatment, potentially indicating an increased filtration of ETP in patients treated with CANA. However, as there was no correlation between the change in ETP in plasma and urine, the increase in U-ETP may be caused by a reduced tubular reabsorption of ETP from urine caused by inhibition of the SGLT2 co-transporter.

Previous studies have shown that COL VI is upregulated in kidneys of patients with renal disease [23], in the myocardium [24], in atherosclerotic lesions [25], heart failure [26], and in cirrhotic liver disease [27]. In a previously published study, a mechanistic link between COL VI formation and poor prognosis was suggested [28]. It was shown that COL VI instigated cardiac fibroblast differentiation in vitro [28]. Furthermore, after induction of myocardial infarction, a marked colocalization between increased COL VI and myofibroblasts was observed in the myocardium of rats [28]. It was therefore suggested that increased COL VI further aggravates fibrosis by driving fibroblast differentiation. Finally, as deficiency of the COL VI alpha-1 chain reduced the damage following myocardial infarction [29], this indicates that the upregulation of COL VI may exacerbate injury. Combined this lends support to the notion that COL VI is not only a consequence, but also a potential cause of disease progression, hence reflecting a poor prognosis.

The involvement of COL VI in kidney disease has mainly been investigated by studies utilizing immunohistochemical analysis [23]. Increased tissue levels of COL VI were prognostic for disease progression in patients with idiopathic membranous nephropathy [30], and increased levels of circulating COL VI were diagnostic for kidney disease [31]. COL VI has recently been under the spotlight due to the discovery and characterization of a bioactive fragment, endotrophin, generated by the cleavage of the C-terminal C5 domain in the α3 chain, during collagen maturation and deposition [18, 19]. Endotrophin has been shown to possess both pro-inflammatory and pro-fibrotic properties. Amongst other properties it enhances TGF-β signaling, attracts macrophages, and promotes epithelial-to-mesenchymal transition [16, 17]. Different studies have shown that overexpression of endotrophin may play an important role in development of non-alcoholic steatohepatitis and hepatocellular carcinoma [32] and that it aggravates cancer invasiveness [16, 33]. The PRO-C6 assay detects levels of circulating and excreted endotrophin. We have previously shown that higher levels of circulating ETP identified a subpopulation of type 2 diabetes patients that responded to insulin sensitizing treatment [34] and that ETP in both serum and urine was independently associated with adverse outcomes in both CKD with mixed etiology [35], in patients with type 1 diabetes [20], and type 2 diabetes [21]. The findings of the manuscript are in line with previous findings for ETP [20, 21, 35, 36]. Our hypothesis is that ETP reflects a pathophysiological alteration of the ECM associated with disease activity and progression, and that it may itself further exacerbate the disease.

Obesity is a leading contributor to morbidity and mortality worldwide amongst others due to promoting cardiometabolic disease and fibrosis [37]. It has been shown that COL VI is also upregulated in dysfunctional adipose tissue, which is characterized by chronic low-grade inflammation that spreads to several tissues and eventually causes cardiovascular disease [37]. In the investigated patients of the CANVAS study, patients ranged from normal weight to high-risk (Class 3 obesity), with the average patient being graded as obese in both the PBO and CANA treated arms. Despite a weak positive association with BMI, the adjustment for BMI did not affect the association of ETP with outcome. Hence, the contribution of the adipose tissue to the pool of circulating ETP may not be substantial, and levels of ETP in circulation may predominantly originate from the kidney and the cardiovascular system.

One of the key predictors and drivers of kidney disease is tubulointerstitial fibrosis, which drives the progressive loss of filtration surface area [10]. As the loss of functional units in the kidney is irreversible, biomarkers reflecting early pathological alterations to the tissue may be superior to biomarkers reflecting functional changes [13]. The investigated patients are predominantly not considered to have kidney disease. It is therefore interesting that ETP is able to independently predict most of the investigated outcomes, as our findings further support that ETP may be an early marker of pathological alterations in the tissue.

Interestingly, the change in P-ETP (ΔP-ETP) was independently associated with all kidney composite endpoints, which may indicate that P-ETP could be used to monitor the risk of kidney endpoints. Of note, the increase in P-ETP over the 3 year period was greater in patients with higher baseline albuminuria. As patients with severely increased albuminuria are known to have a worse disease trajectory, the higher increase in P-ETP in these patients may indicate that COL VI formation and endotrophin release is higher in these patients. Our findings support previously published studies showing that ETP is associated with outcomes, a phenomenon that is independent of albuminuria, and that it adds incremental predictive value [20, 21, 35, 36]. Despite the lack of impact of treatment on levels of P-ETP, we found that an increase in P-ETP in the placebo arm was independently associated with all-cause mortality. This may indicate that P-ETP could also be used to monitor risk of all-cause mortality. Further studies need to be conducted to verify these findings.

Our bootstrap analyses revealed, that even in the presence of known risk factors (e.g., NTproBNP, prior history of HF and CV disease etc.), P-ETP was still consistently retained in the models predicting the outcomes. This indicates that P-ETP reflects a novel process that can be used to further the understanding of the pathology driving these outcomes.

The strengths of this study were that ETP was measured in the CANVAS study, in which patients were monitored according to predefined protocols living up to the highest quality standards, and the analyses were adjusted for important risk factors selected based on unbiased analysis (i.e., AIC). However, the comprehensive adjustments for the risk factors in conjunction with the relatively low number of events for some of the outcomes may lead to unstable model estimates due to overfitting. The patients in this study are generally in early disease stages, and it is therefore interesting to see that ETP is able to independently predict outcomes in these patients, where it is less likely that irreversible alterations of the tissue architecture have taken place. Intervention in patients with early disease are most likely to have the greatest impact as their parenchyma is still functional.

One of the limitations of the study is that we are not able to provide causal evidence for the increased risk of investigated outcomes in patients with higher P-ETP. ETP was a better prognostic marker in plasma than in urine. Levels of P-ETP may therefore reflect the overall systemic levels of COL VI, rather than reflecting specific changes in the kidney, which may be more apparent from urine levels. Two additional limitations are the potential under adjustment of biomarker associations due to imprecisely measured covariates, and a limited power for the subgroup analysis (e.g., due to few events, or few patients in the investigated groups).

## Conclusions

Levels of ETP predict relevant clinical outcomes in patients with diabetes. Based on these and previously published findings for ETP, we believe that ETP merits further investigation as a risk marker in patients with diabetes. In the future, it would be important to determine whether changes in ETP predict response to kidney- and/or cardioprotective therapies.

## Supplementary Information

**Additional file 1: Figure 1.** Hazard ratios for tertiles of P-ETP at baseline. Patients were stratified into tertiles based on P-ETP levels at baseline. Unadjusted associations of P-ETP tertiles with outcome are depicted as HR [95% CI]. **Figure 2.** Association of U-ETP with outcome. Patients were stratified based on baseline U-ETP into tertiles and Kaplan-Meier curves were plotted for each tertile. Risk was assessed with reference to tertile 1 for A) heart failure (HF), B) CV death, C) the composite of HF and CV death, D) all-cause mortality, E) the kidney composite endpoint 1 (KCOM1; 40% decrease in eGFR, kidney death, or ESKD), F) -2 (KCOM2; KCOM1 and CVD), and G) -3 (KCOM3; KCOM1 and conversion to severely increased albuminuria).

## Data Availability

Data is available upon reasonable request. Data can only be used for non-commercial purposes, and only as long as participant confidentiality is not breached.

## Abbreviations

ACR: Albumin:creatinine ratio
CANA: Canagliflozin
CANVAS: CANagliflozin cardioVascular Assessment Study
CV: Cardiovascular
ECM: Extracellular matrix
HFCVD: Composite of HF and CVD (HFCVD)
KCOM1: Composite kidney endpoint defined as a sustained 40% decline of eGFR, end-stage kidney disease defined as an eGFR < 15 mL/min/1.73 m^2^, need for dialysis or kidney transplantation, or kidney death
KCOM2: Kidney composite endpoint including KCOM1 and CVD
KCOM3: Kidney composite endpoint including KCOM1 and conversion to severely increased albuminuria
PBO: Placebo
P-ETP: Plasma endotrophin
U-ETP: Urinary endotrophin
