# Supplementary material for: Endotrophin is a risk marker of complications in CANagliflozin cardioVascular Assessment Study (CANVAS): a randomized controlled trial
Source: Cardiovasc Diabetol. 2022 Nov 28;21:261. doi: 10.1186/s12933-022-01666-7 (PMC9706889; doi:10.1186/s12933-022-01666-7)
Supplement: Supplementary file 1 — Additional file 1: Figure 1. Hazard ratios for tertiles of P-ETP at baseline. Patients were stratified into tertiles based on P-ETP levels at baseline. Unadjusted associations of P-ETP tertiles with outcome are depicted as HR [95% CI]. Figure 2. Association of U-ETP with outcome. Patients were stratified based on baseline U-ETP into tertiles and Kaplan-Meier curves were plotted for each tertile. Risk was assessed with reference to tertile 1 for A) heart failure (HF), B) CV death, C) the composite of HF and CV death, D) all-cause mortality, E) the kidney composite endpoint 1 (KCOM1; 40% decrease in eGFR, kidney death, or ESKD), F) -2 (KCOM2; KCOM1 and CVD), and G) -3 (KCOM3; KCOM1 and conversion to severely increased albuminuria). [file 12933_2022_1666_MOESM1_ESM.docx]

## Supplemental tables and figures

**Supplemental Figure 1**. **Hazard ratios for tertiles of P-ETP at baseline.** Patients were stratified into tertiles based on P-ETP levels at baseline. Unadjusted associations of P-ETP tertiles with outcome are depicted as HR [95% CI].

**Supplemental Figure 2.** **Association of U-ETP with outcome**. Patients were stratified based on baseline U-ETP into tertiles and Kaplan-Meier curves were plotted for each tertile. Risk was assessed with reference to tertile 1 for A) heart failure (HF), B) CV death, C) the composite of HF and CV death, D) all-cause mortality, E) the kidney composite endpoint 1 (KCOM1; 40% decrease in eGFR, kidney death, or ESKD), F) -2 (KCOM2; KCOM1 and CVD), and G) -3 (KCOM3; KCOM1 and conversion to severely increased albuminuria).
